# Novel Photoinduced Squalene Cyclic Peroxide Identified, Detected, and Quantified in Human Skin Surface Lipids

**DOI:** 10.3390/antiox10111760

**Published:** 2021-11-04

**Authors:** Saoussane Khalifa, Masaru Enomoto, Shunji Kato, Kiyotaka Nakagawa

**Affiliations:** 1 Food and Biodynamic Chemistry Laboratory, Graduate School of Agricultural Science, Tohoku University, Sendai 980-8577, Japan; khalifa.saoussane.q3@dc.tohoku.ac.jp (S.K.); shunji.kato.b5@tohoku.ac.jp (S.K.); 2 Applied Bioorganic Chemistry Laboratory, Graduate School of Agricultural Science, Tohoku University, Sendai 980-8577, Japan; masaru.enomoto.a2@tohoku.ac.jp

**Keywords:** SQ, SQ-OOH, cyclic peroxides, photooxidation, ^1^O_2_, ^3^O_2_, peroxyl radical, 2-OOH-3-(1,2-dioxane)-SQ, SSLs

## Abstract

Skin surface lipids (SSLs) form the first barrier that protects the human organism from external stressors, disruption of the homeostasis of SSLs can result in severe skin abnormalities. One of the main causes of this disruption is oxidative stress that is primarily due to SSLs oxidation. Squalene (SQ), the most abundant lipid among SSLs, was shown to first undergo singlet molecular oxygen (^1^O_2_) oxidation to yield 6 SQ-monohydroperoxide (SQ-OOH) isomers as the primary oxidation products. However, due to the instability and lability of hydroperoxides, we found that when total SQ-OOH isomers are further photooxidized, they form a unique higher molecular weight secondary oxidation product. To generate the compound, we photooxidized total SQ-OOH isomers in the presence of ground state molecular oxygen (^3^O_2_), after its isolation and purification, we studied its structure using MS/MS, NMR, derivatization reactions, and chemical calculations. The compound was identified as 2-OOH-3-(1,2-dioxane)-SQ. Photooxidation of individual SQ-OOH isomers revealed that 6-OOH-SQ is the precursor of 2-OOH-3-(1,2-dioxane)-SQ and indicated the possibility of the formation of similar cyclic peroxides from each isomer following the same photoinduced chain reaction mechanism. An HPLC-MS/MS method was developed for the analysis of 2-OOH-3-(1,2-dioxane)-SQ and its presence on the skin was confirmed in SSLs of six healthy individuals. Its quantity on the skin correlated directly to that of SQ and was not inversely proportional to its precursor, indicating the possibility of its accumulation on the skin surface and the constant regeneration of 6-OOH-SQ from SQ’s oxidation. In general, research on lipid cyclic peroxides in the human organism is very limited, and especially on the skin. This study shows for the first time the identification and presence of a novel SQ cyclic peroxide “2-OOH-3-(1,2-dioxane)-SQ” in SSLs, shedding light on the importance of further studying its effect and role on the skin.

## 1. Introduction

**Figure 1:**
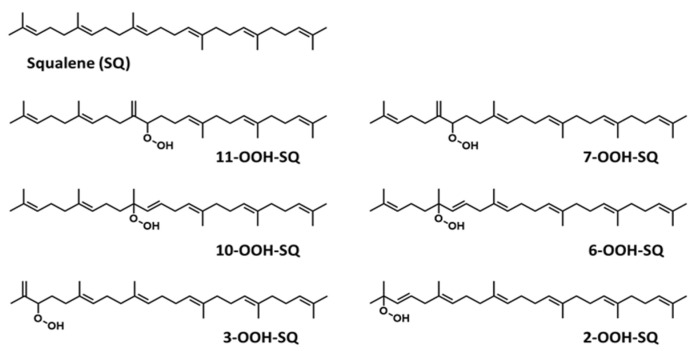
Chemical structure of unoxidized squalene (SQ) and its six singlet oxygen monohydroperoxide products (SQ-OOH isomers).

The skin forms the first defense line that protects the organism against external stressors and pathogens due to its hydrophobic coating with skin surface lipids (SSLs) [1]. Dysregulation of SSLs’ function has been linked to their oxidation resulting in serious skin conditions such as premature aging, melanoma, inflammatory and allergic diseases [2,3,4]. For instance, malondialdehyde (MDA) has been shown to be involved in cell ferroptosis induced in melanoma [5]. Moreover, skin biopsies from psoriatic patients showed an elevated level of 9- and 13-hydroxy-octadecadienoic acids as well as 8- and 12-hydroxy-eicosatetraenoic acids [6]. Squalene (SQ) (SQ, Figure 1), the most abundant among SSLs (12%), and a precursor of natural steroids [7,8,9], plays an overall protective role on the skin, as it acts as an antioxidant and a protective barrier [10,11], it also enhances the skin’s immunity through the promotion of IL-1β and the activation of T cells [12,13]. SQ is also often consumed as a supplement extracted from shark liver oil (its richest natural source) [14], or as a component of different edible oils [15], it exhibits multiple health benefits from being an antioxidant [10,16], a nanoparticle to deliver therapeutic agents [17,18], an adjuvant [19,20,21], and an anticancer agent [22].

Over the years, lipid hydroperoxides and their mechanism of formation have been extensively studied as primary products of lipid oxidation, and they have long been linked to different pathogenesis such as accelerated aging, and inflammatory diseases [23,24,25]. Similarly, due to the terpenic nature of SQ, it has been reported to undergo singlet oxygen (^1^O_2_) oxidation on the skin to yield six SQ-monohydroperoxides (SQ-OOH) isomers as its primary oxidation products (Figure 1) [26]. Previously, we demonstrated that the six SQ-OOH isomers increased following the exposure of the skin to sunlight, further confirming the photosensitized ^1^O_2_ oxidation of SQ [27]. Its oxidation on the skin can cause significant abnormalities such as hyperpigmentation, inflammation, and formation of wrinkles [28,29,30,31]. However, studies on its secondary oxidation products and the mechanism involved in their formation are rather limited to its breakdown products, mainly upon interaction with ozone [32,33,34]; no significant reports on the formation of higher molecular weight secondary oxidation products could be found. Taking into account the constant exposure of the skin to photons (e.g., sunlight, LEDs), and the unstable nature of hydroperoxides, we assumed that SQ-OOH isomers would undergo further modifications to yield cyclic peroxides as the first secondary oxidation products.

The aim of this study is to verify the formation of higher molecular weight cyclic peroxides as secondary oxidation products resulting from SQ-OOH isomers, and to confirm their presence on the skin. To this end, we photooxidized SQ-OOH isomers as a mixture in the presence of ground state molecular oxygen (^3^O_2_). The resulting main secondary oxidation product was isolated, and its precursor and structure were determined using MS/MS, NMR, derivatization reactions, and chemical calculations. Furthermore, oxidation of individual SQ-OOH isomers was carried out to investigate if similar products could be formed from each SQ-OOH isomer. Lastly, we developed an HPLC-MS/MS method for the newly identified compound and checked its presence and quantities in human SSLs of six healthy volunteers in comparison to unoxidized SQ, and SQ-OOH isomers from the same subjects. The results obtained herein offer a new perspective into the oxidation products and mechanism that can be implicated in the skin’s oxidative state, as well as the possible use of the newly described secondary oxidation product as a skin’s oxidative stress biomarker. Such results also open new doors to investigate antioxidants targeting the prevention and scavenging of the described compound, as well as to study its effect and role on the skin.

## 2. Materials and Methods

### 2.1. Materials

SQ (99%), RB, 2-methoxypropene (SSL), and NaIO_4_ were obtained from FUJIFILM Wako Pure Chemical (Osaka, Japan). PPTS and zinc dust were purchased from Sigma Aldrich, Inc. (St. Louis, MO, USA). All other reagents were of the highest grade available.

### 2.2. Preparation of SQ-OOH Isomers

SQ (30 g) was dissolved in ethanol (100 mg/mL), photooxidized (^1^O_2_ oxidation) using RB as a type II photosensitizer (PSII) (at a final concentration of 0.01 mg/mL) under an LED light of 55 Klux intensity at 4 °C with constant stirring. After 7 h of oxidation, RB was removed using a QMA cartridge (12 cc Waters, Milford, MA, USA) following our previously described method [27]. To ensure the formation of SQ-OOH, 10 µL of the resulting solution was evaporated and diluted in hexane (1 mg/mL) for NP UV-HPLC analysis at 210 nm using a silica column (Inertsil SIL 100A 5 μm, 4.6 × 250 mm, GL Sciences, Tokyo, Japan) maintained at 40 °C and a mobile phase consisting of hexane/isopropyl alcohol (100:0.5) at a flow rate of 1 mL/min.

The generated SQ-OOH isomers and unoxidized SQ were separated by RP semi-preparative HPLC using a COSMOSIL 5C_18_-MS-Ⅱ (20 × 250 mm, NACALAI TESQUE, Inc., Kyoto, Japan) column maintained at 40 °C, and 100% methanol as the mobile phase at a flow rate of 12 mL/min. A portion of the obtained SQ-OOH (isomers mixture) was purified by NP semi-preparative HPLC to obtain individual SQ-OOH isomers, each isomer was reacted with Mxp to protect the hydroperoxide group, purified then deprotected using PPTS followed by several purifications as described previously [35].

The SQ-OOH (isomers mixture) and individual SQ-OOH isomers were analyzed by NP-UV-HPLC as described above then dissolved in ethanol and stored in the dark under nitrogen gas at −80 °C until use.

### 2.3. Photooxidation of SQ-OOH Isomers

A mixture of SQ-OOH isomers was dissolved in hexane at a concentration of 15 mg/mL and exposed to an LED light of 65 Klux intensity with constant stirring at 4 °C in the presence of ^3^O_2_ and absence of a photosensitizer. After 12 h of oxidation, the solution was analyzed by NP-UV-HPLC as stated above. A new peak was detected at around 21.9 min. The newly observed compound was collected using silica-based open column chromatography eluted with hexane/ethyl acetate (90:10) linked to a UV detector at 210 nm and a pump operating at 12 mL/min. The obtained compound was subsequently purified several times using the previously mentioned semi-preparative HPLC systems in both NP and RP. The pure compound was stored in ethanol in the dark at −80 °C under nitrogen gas until further analysis.

### 2.4. Structural Elucidation of the Purified Secondary Oxidation Product and Oxidation of Individual SQ-OOH Isomers

To elucidate the structure of the isolated and purified compound, Q1 scan and PIS (ESI, positive mode) were carried out on a micrOTOF-Q II (Bruker Daltonik, GmbH, Bremen, Germany) in the presence of an alkali metal (Na^+^), as it was proven efficient in the characteristic fragmentation of lipid hydroperoxides in our previous studies [36,37,38]. Na^+^ was used since it will most likely produce characteristic fragments from both hydroperoxides and cyclic peroxides. In addition, to determine the exact SQ-OOH isomer precursor that generated the newly observed secondary oxidation product, the SQ-OOH isomers that were noticed to disappear were oxidized individually in the conditions mentioned above, Q1 scan and PIS were carried out on their oxidation products and compared to that of the newly obtained secondary oxidation product.

The structure of the isolated and purified compound was further studied through several procedures. ^1^H, ^13^C, DEPT and 2D, nuclear magnetic resonance (NMR) (heteronuclear multiple bond correlation (HMBC), heteronuclear single quantum coherence (HSQC), correlation spectroscopy (COSY), nuclear Overhauser effect spectroscopy (NOESY)) were recorded on a Varian NMR (Palo Alto, CA, USA) Unity 600TT spectrometer at 600 MHz using deuterated chloroform (CDCl_3_) as a solvent and tetramethylsilane (TMS) as the reference. A total of 4 candidate structures were hypothesized, among which the final structure was confirmed by a 2-step derivatization reaction and chemical calculations using Spartan’18 (Wavefunction Inc., Irvine, CA, USA) software. The first step of the derivatization reaction consisted of a reduction using zinc dust (50 equivalent, in acetic acid/dichloromethane (1:2)) [39], the product was subsequently purified using RP semi-preparative HPLC in the conditions mentioned before, the second step consisted of oxidative cleavage of vicinal alcohols using sodium periodate (NaIO_4_) (3.1 equivalent) in acetonitrile/H_2_O (3:1). The products of this reaction were confirmed by Q1 scan (Supporting Information I, Figure S2). The chemical calculations of the ^13^C chemical shifts of all possible conformers were compared to the experimental values using Boltzmann distribution and ωB97X-V as the mathematical model [40].

After determining the structure, a detailed study of the Q1 scan and PIS spectra (conditions can be found in Supporting Information I, Table S2) of the individually oxidized SQ-OOH isomers was carried out in the same oxidation conditions, however, due to the scarce quantities of pure individual SQ-OOH isomers, 200 µg/200 µL of hexane was photooxidized of each isomer. One main secondary oxidation product was detected and its structure was proposed in each case. A reaction with Mxp was conducted to verify the suggested structures. Due to the scarce quantities of individual SQ-OOH isomers, pure standards of their respective new secondary oxidation products could not be purified in considerable amounts for detection and quantification from SSLs.

### 2.5. Analysis of the Identified Compound from Human SSLs

An HPLC-MS/MS method was first developed to analyze the newly identified compound on a 4000 quadrupole/linear ion-trap tandem mass spectrometer (4000QTRAP) (SCIEX, Tokyo, Japan) using a COSMOSIL 5C_18_-MS-Ⅱ (2.1 × 250 mm, NACALAI TESQUE, INC., Kyoto, Japan) column with methanol 100% as the mobile phase at a flow rate of 200 µL/min (MS/MS conditions can be found in Table S1 of Supporting Information I).

SSLs were extracted from the forehead area of 6 healthy volunteers according to our previously described method with modification [27]. All participants gave informed consent prior to sampling, the experiments were conducted in accordance with the Declaration of Helsinki, and the protocols were approved by the ethics committee of Tohoku University Graduate School of Agricultural Science (registration numbers: 19-A-06). In brief, medical cotton swabs were soaked in acetone for 15 min several times, the middle forehead area was then wiped with the swab about 10 times. The tip of the swab was collected into an amber vial containing 10 mL of acetone/chloroform (1:1), vortexed for 10 min then centrifuged for 10 min at 2000× *g*. This operation was repeated twice, and the fractions were combined and evaporated under nitrogen gas (using a spin dryer). The obtained sample was dissolved in 1 mL methanol/methyl *tert*-butyl ether (1:2), 50 µL of this sample was collected for SQ analysis using an HPLC-MS/MS method reported previously [41]. The rest of the fraction was evaporated and dissolved in 70% methanol and purified using an equilibrated C18 cartridge (Strata C18-E, 55 µm, 70 A, 100 mg, 1 mL. Phenomenex, Torrance, CA, USA) [26]. The obtained eluate was evaporated then dissolved in 950 µL hexane. In total, 150 µL of this fraction was evaporated then dissolved in methanol for the analysis of the new SQ secondary oxidation product, while the remaining 800 µL was used to analyze SQ-OOH isomers using a previously reported HPLC-MS/MS method [42]. The concentrations of individual SQ-OOH standards and the secondary oxidation product were determined prior to HPLC-MS/MS analyses using the ferrous oxidation-xylenol orange (FOX) assay as described previously [43,44,45], except in the case of 10-OOH-SQ where it was gravimetrically weighed due to its extreme lability to the assay.

## 3. Results and Discussion

### 3.1. Preparation of Total and Individual SQ-OOH Isomers

**Figure 2:**
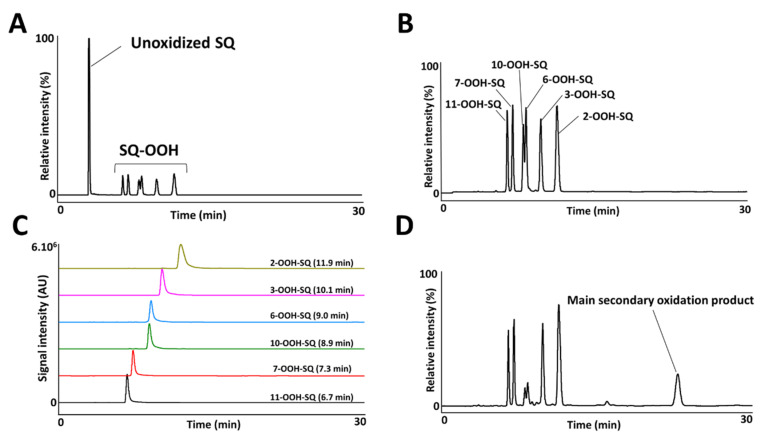
UV-HPLC chromatograms of the crude oxidized SQ (**A**), purified mixture of SQ-OOH isomers (**B**), purified individual SQ-OOH isomers (**C**), and the photooxidized mixture of SQ-OOH isomers (**D**).

The photooxidation of SQ in the presence of RB (PSII) proceeded by an ene reaction following ^1^O_2_ generation by the photoexcited RB from atmospheric ^3^O_2_, and gave 6 SQ-OOH isomers with approximately equal amounts and unoxidized SQ as shown in Figure 2A. Peaks of the isolated and purified fraction of SQ-OOH isomers as shown in Figure 2B were identified from 1 to 6 as follows: 11-OOH-SQ, 7-OOH-SQ, 10-OOH-SQ, 6-OOH-SQ, 3-OOH-SQ, 2-OOH-SQ. These results were consistent with previous reports [27,35]. Individual SQ-OOH isomers were isolated and purified with a purity of at least 98% (Figure 2C). Their purity was verified by UV-HPLC and HPLC-MS/MS (data not shown).

### 3.2. Photooxidation of SQ-OOH Isomers

**Figure 3:**
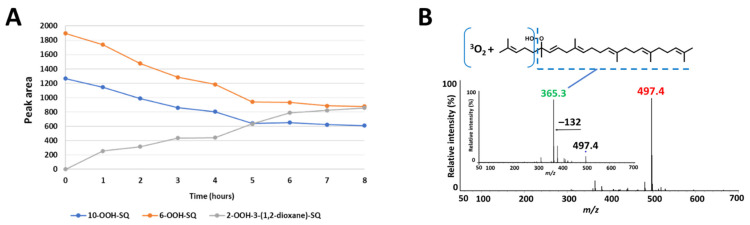
(**A**) correlation between the decrease in 10-OOH-SQ, 6-OOH-SQ and the increase in 2-OOH-3-(1,2-dioxane)-SQ. The peak area of 6-OOH-SQ decreased by a value of 1019 while 2-OOH-3-(1,2-dioxane)-SQ’s peak area reached 856 after 8 h, equal to 84% of the decreased 6-OOH-SQ. (**B**) Q1 and product ion scan (PIS) spectra of the newly observed SQ secondary oxidation product.

Upon exposure of SQ-OOH isomers to photons, and in the presence of ^3^O_2_, the profile of SQ-OOH isomers following NP UV-HPLC analysis showed the disappearance of the two isomers: 10-OOH-SQ and 6-OOH-SQ, with the appearance of one prominent peak at 21.9 min (Figure 2D). The decrease in 10-OOH-SQ and 6-OOH-SQ correlated with the appearance of the new secondary oxidation product time-dependently (Figure 3A). We assumed that the precursor of the observed peak is either 10-OOH-SQ or 6-OOH-SQ. The new compound was successfully collected and purified upon open column chromatography and semi-preparative HPLC in both NP and RP with a purity equal to 99% and a quantity of 425 mg. The targeted oxidation of 6-OOH-SQ and 10-OOH-SQ in the mixture of the six isomers will be explained along with the mechanism in the upcoming section.

### 3.3. Structural Elucidation of the Purified Secondary Oxidation Product

Q1 scan of the newly collected secondary oxidation product revealed *m/z* 497 = [(SQ-OOH + O_2_) + Na]^+^, its PIS showed one main fragment with *m/z* 365 (Figure 3B). Previously, we assumed that either 10-OOH-SQ or 6-OOH-SQ is the precursor of the new secondary oxidation product; to this end, we photooxidized 6-OOH-SQ and 10-OOH-SQ individually and scanned their oxidation products using Q1 scan. We found that unlike 10-OOH-SQ (Supporting Information I, Figure S1A), 6-OOH-SQ produced one main secondary oxidation product with the same *m/z* as the newly collected peak (*m/z* 497). Its PIS showed the exact same fragmentation (*m/z* 365) observed upon PIS of the newly collected compound (Supporting Information I, Figure S1B). This fragmentation is also observed on the precursor, unoxidized 6-OOH-SQ (Supporting Information I, Figure S1C), indicating the incorporation of ^3^O_2_ in the lost fragment of the molecule (Figure 3B). These observations can be interpreted by the possibility of the presence of a cyclic peroxide on this fragment.

**Figure 4:**
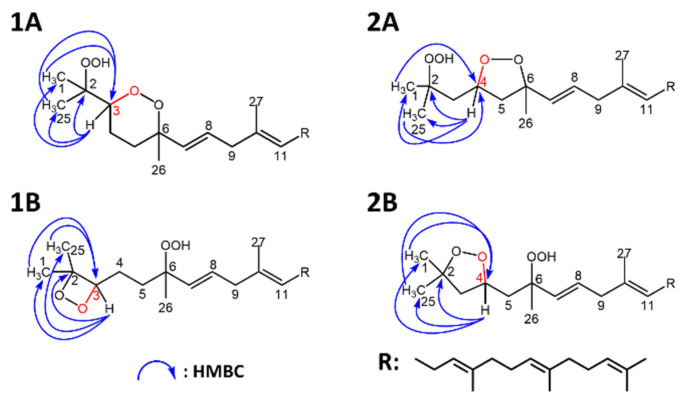
Candidate structures of SQ secondary oxidation product and their heteronuclear single quantum coherence (HMBC) correlations. (**1A**,**1B**), based on the correlations of H1 and H25 to C3, and H3 to C1, C2 and C25; (**2A**,**2B**), based on the correlations of H1 and H25 to C4, and H4 to C1, C2 and C25.

**Figure 5:**
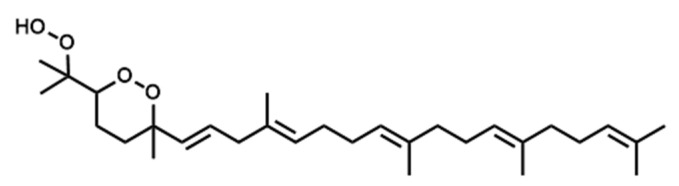
Chemical structure of the newly identified SQ secondary oxidation product, 2-OOH-3-(1,2-dioxane)-SQ.

To confirm this, ^1^H NMR showed characteristic peaks (δH 4.22) that were absent from the ^1^H NMR spectrum of 6-OOH-SQ corresponding to either H3 or H4. Four candidate structures (Figure 4) were hypothesized upon observation of HMBC characteristic correlations (H3 (1A, 1B) or H4 (2A, 2B) to C1, C25 and C2; H1 and H25 to C3 (1A, 1B) or C4 (2A, 2B)). (Full ^1^H and ^13^C assignments, 1D and 2D spectra can be found in the Supporting Information I, Section S8). To determine the exact chemical structure among the four possibilities, first, a 2-step derivatization reaction was carried out. Treatment of the compound with zinc dust resulted in a triol that would bear vicinal alcohols in the case of structures 1A and 1B and non-vicinal alcohols in the case of 2A and 2B. After purification of the triol, it was reacted with NaIO_4_ that selectively targets vicinal alcohols through oxidative cleavage. The reaction with NaIO_4_ proceeded yielding one main fragment as observed by Q1 scan (Supporting Information I, Figure S2) that could be generated only from 1A or 1B. Next, calculations of the ^13^C chemical shifts using Boltzmann distribution (ωB97X-V was used as the mathematical model) in Spartan’18 (Wavefunction Inc.) software gave a max absolute value inferior to 2 and a low RMS value in case of structure 1A in its cis configuration, whereas this value was higher than 2 with a higher RMS value in all of the other possible structures (Table 1). The calculations method followed here [40] indicates that the structure of the compound is most compatible with 1A in its cis configuration. Although its trans isomer had a max absolute value equal to 3.4, it is possible that it exists as a minor configurational isomer of the compound following a detailed interpretation of the NMR data (Supporting Information I, Table S5) (detailed calculations can be found in the Supporting Information II). Based on all of the aforementioned results, the structure of the newly observed compound was determined to be 2-OOH-3-(1,2-dioxane)-SQ mainly in its cis configuration (Figure 5). To the best of our knowledge, this is the first report showing the formation of a cyclic peroxide from SQ in general and upon photooxidation in particular.

**Figure 6:**
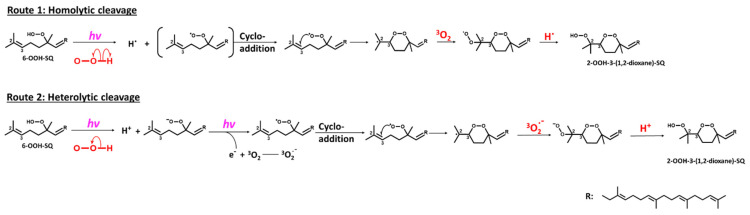
Proposed mechanism involved in the formation of 2-OOH-3-(1,2-dioxane)-SQ from 6-OOH-SQ following two possible routes.

With regard to the oxidation mechanism involved in the formation of 2-OOH-3-(1,2-dioxane)-SQ from 6-OOH-SQ, we hypothesized that upon exposure of 6-OOH-SQ to photons, a peroxyl radical is formed from the hydroperoxide group either directly, by homolytic cleavage of the O-H bond to give a peroxyl radical and a hydrogen radical, or indirectly by heterolytic cleavage yielding a peroxyl anion and a cation. Further exposure of the peroxyl anion to photons results in the release of a photoelectron that reacts subsequently with ^3^O_2_ to give a superoxide anion (^3^O_2_^−^) and a peroxyl radical. In both possibilities, the peroxyl radical forms a cyclic peroxide (1,2-dioxane) through endo-cycloaddition on C3 leaving a radical on C2. In the first case, the radical readily reacts with ^3^O_2_ to yield a peroxyl radical on C2 then a hydroperoxide upon reaction with the hydrogen radical. In the second possible route, the superoxide anion forms a peroxyl anion on C2 which reacts with the cation released from the heterolytic cleavage to give a hydroperoxide (Figure 6). Although, several reports showed the formation of a hydroxyl radical and an alkoxy radical upon further oxidation of alkenes’ hydroperoxides under various conditions [46,47,48,49], different from ours, due to the lower dissociation energy of the group’s O-O bond compared to that of the O-H bond. These dissociation energies, however, are basically the homolytic cleavage energies of the bonds under thermal conditions, which are based on the triplet excitation energy of the σ bond. On the other hand, the heterolytic cleavage energies are rarely taken into consideration and they certainly vary from the known bond dissociation energies (BDEs) as they are based on the singlet excitation energy of the σ bond. In conclusion, when it comes to heterolytic cleavage, and under the present conditions (*hv* + ^3^O_2_), common O-O and O-H BDEs cannot be used to predict the products that can be formed. Additionally, classic O-O homolytic cleavage would have produced a rather considerable amount of hydroxyl radicals, these reactive species would have formed mostly water molecules by hydrogen abstraction either from adjacent hydroperoxides or from other carbons [47]. As the used solvent was hexane and the oxidized SQ-OOH quantity was of the order of grams, a clear non-miscible aqueous phase would have formed, an observation that did not take place in our study. In addition, the amount of the generated 2-OOH-3-(1,2-dioxane)-SQ was equal to 84% of the decreased 6-OOH-SQ, indicating the formation of one main intermediate radical (i.e., peroxyl radical and not alkoxy radical) leading to one main final product (2-OOH-3-(1,2-dioxane)-SQ) (Figure 3A). Based on all of the above evidence, we think that the second route of the proposed mechanism may be the most probable.

This also explains the targeted oxidation of 6-OOH-SQ and 10-OOH-SQ in the mixture of the six isomers, which is most likely due to the fact that they both bear tertiary hydroperoxides, increasing the overall electron density due to the inductive effect, making the reactivity, electron transfer, and photolysis more probable in a competitive system such as the present with four other isomers with different properties. Although 2-OOH-SQ also bears a tertiary hydroperoxide, the presence of a double bond on C3 would make its first secondary intermediate oxidation product quite unstable (1,2-dioxetane), giving priority to 6-OOH-SQ and 10-OOH-SQ to interact first with photons and oxygen in the mixture.

### 3.4. Oxidation of Individual SQ-OOH Isomers

**Figure 7:**
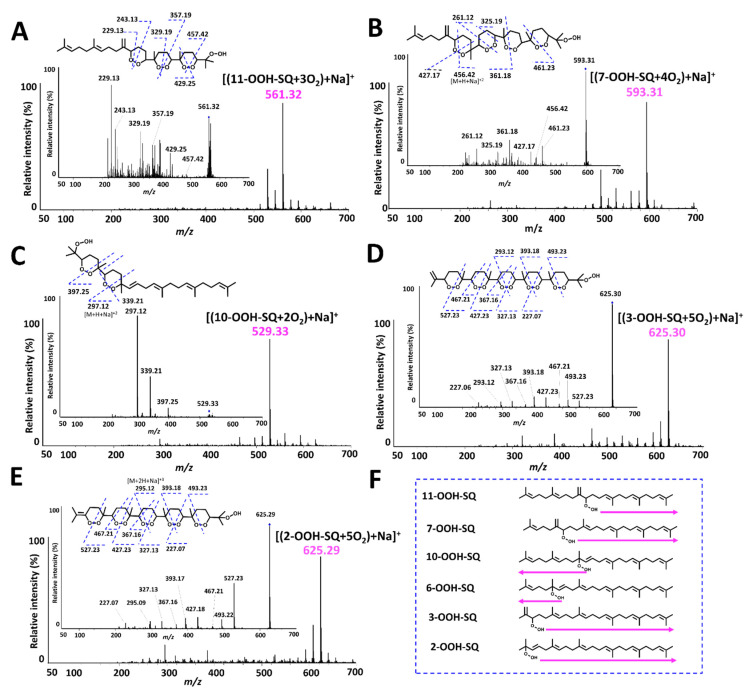
Q1 and PIS spectra of the main photooxidation products of individual SQ-OOH isomers and their suggested chemical structures. (**A**) [(11-OOH-SQ + 3O_2_) +Na]^+^; (**B**) [(7-OOH-SQ + 4O_2_) +Na]^+^; (**C**) [(10-OOH-SQ + 2O_2_) +Na]^+^; (**D**) [(3-OOH-SQ + 5O_2_) +Na]^+^; (**E**) [(2-OOH-SQ + 5O_2_) +Na]^+^; (**F**) direction of the chain reaction’s propagation.

Photooxidation of individual SQ-OOH isomers gave one main secondary oxidation product among other minor by-products upon Q1 scan (Figure 7). All main secondary oxidation products showed a pattern in the number of incorporated ^3^O_2_ that is compatible with the oxidation mechanism proposed for the formation of 2-OOH-3-(1,2-dioxane)-SQ from 6-OOH-SQ. For instance, 11-OOH-SQ gave a product of *m/z* 561 = [(11-OOH-SQ + 3O_2_) + Na]^+^, 7-OOH-SQ gave a product of *m/z* 593 = [(7-OOH-SQ + 4O_2_) + Na]^+^, 10-OOH-SQ, gave a product of *m/z* 529 = [(10-OOH-SQ + 2O_2_) + Na]^+^. For 3-OOH-SQ and 2-OOH-SQ, they both gave a main secondary oxidation product of *m/z* 625 = [(SQ-OOH + 5O_2_) + Na]^+^. In each case, the number of incorporated ^3^O_2_ was equal to the number of unsaturations in the direction indicated by the arrows on Figure 7F on each SQ-OOH isomer, a direction that would produce a more stable cyclic peroxide (1,2-dioxane), as the incorporation of ^3^O_2_ in the opposite direction would result in a 1,2-dioxetane of a relatively unstable nature. However, there was an exception in the case of 2-OOH-SQ, where we suggest that a 1,2-dioxetane is first formed to ultimately give a 1,2-dioxane. Interestingly, the intensity of these compounds increased over a period of 48 h in total (data not shown) except in the case of 10-OOH-SQ where it quickly decomposed when oxidized for more than 4 h (Supporting Information I, Figure S3). A previous report [50] showed the generation of breakdown products from the acid decomposition of methyl linolenate and linoleate cyclic peroxides, however, no reports could be found on the photoinduced decomposition of lipid cyclic peroxides.

Based on the chain reaction mechanism proposed for 2-OOH-3-(1,2-dioxane)-SQ and the Q1 scan of each SQ-OOH isomer’s photooxidation, the main secondary oxidation product in each case is suggested to be bearing several cyclic peroxides (1,2-dioxanes) and a hydroperoxide at the end of the chain (Figure 7). The proposed structure for each secondary oxidation product was confirmed by the fragmentations observed upon PIS (Figure 7). We further confirmed the hypothesized structures by reacting the crude oxidation products of each oxidized SQ-OOH isomer with Mxp, the latter reacts with hydroperoxides to yield a perketal adduct, we assumed that if each main secondary oxidation product forms a single Mxp adduct, it confirms the presence of one hydroperoxide group, and confirms the proposed structures. Effectively, upon Q1 scan, there was one Mxp adduct formed with each main secondary oxidation product confirming further the proposed structures (Supporting Information I, Figure S4). There are very few reports on the formation of lipid cyclic peroxides and they were mostly generated by thermal autooxidation or ^1^O_2_ oxidation of lipids [51,52,53,54], however, to the best of our knowledge, no reports could be found on the generation of lipid cyclic peroxides upon exposure to photons and ^3^O_2_, and no evidence on SQ cyclic peroxides formation both in vitro and in vivo.

### 3.5. Analysis of the Identified Compound from Human SSLs

SSLs, deriving from both sebaceous and epidermal origins, were extracted using acetone-soaked cotton swabs from the forehead area of six healthy volunteers. The lipids collected on the cotton swab gather both polar and non-polar fractions of SSLs. Considering that the majority of SSLs are composed of non-polar lipids, after extraction of the lipids from the cotton swab, SQ, given its non-polar nature was analyzed directly using an HPLC-MS/MS method that we reported previously [41]. Whereas for SQ-OOH and 2-OOH-3-(1,2-dioxane)-SQ, they were analyzed only after purification by solid-phase extraction on a C18 cartridge to decrease the amount of non-polar contaminants. SQ-OOH isomers were analyzed by an HPLC-MS/MS method reported previously [42], whereas for 2-OOH-3-(1,2-dioxane)-SQ, a new HPLC-MS/MS method was developed in RP-HPLC. The quantities of 2-OOH-3-(1,2-dioxane)-SQ, SQ, and SQ-OOH were determined using external calibration curves made by the synthesized standards.

**Figure 8:**
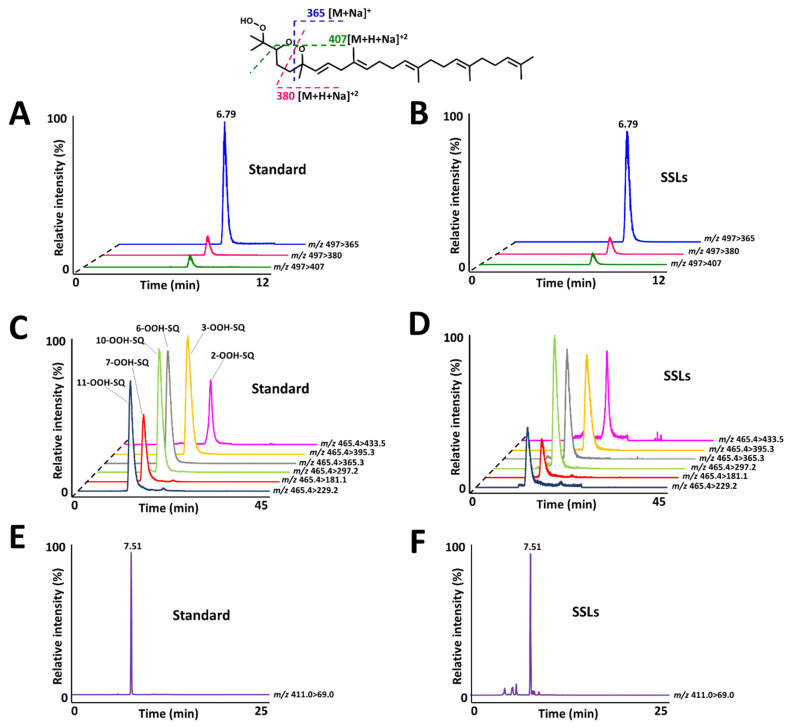
(**A**) 2-OOH-3-(1,2-dioxane)-SQ standard’s chromatogram with characteristic fragments as demonstrated on the structure. (**B**) 2-OOH-3-(1,2-dioxane)-SQ chromatogram from skin surface lipids (SSLs). (**C**) SQ-OOH standards’ chromatogram. (**D**) SQ-OOH chromatogram from SSLs. (**E**) SQ standard’s chromatogram. (**F**) SQ chromatogram from SSLs.

**Figure 9:**
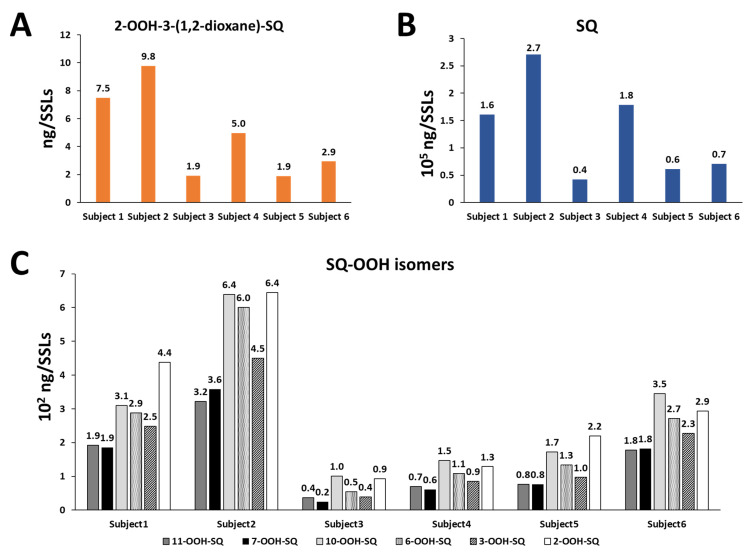
Quantities of 2-OOH-3-(1,2-dioxane)-SQ (**A**), of SQ (**B**), and of SQ-OOH isomers (**C**) in SSLs of six healthy individuals calculated by the use of external standards using HPLC-MS/MS.

2-OOH-3-(1,2-dioxane)-SQ was detected in all tested subjects, the standard’s and SSLs’ chromatograms are presented in Figure 8A,B, with quantities ranging between 1.9 and 9.8 ng per sampled area (Figure 9A). Whereas, the quantities of its precursor, 6-OOH-SQ, ranged between 0.5 × 10^2^ and 6.0 × 10^2^ ng per sampled area (Figure 9C). The chromatograms of SQ-OOH further confirmed the ^1^O_2_ oxidation of SQ in SSLs (Figure 8C,D) and the percentage of 6-OOH-SQ among SQ-OOH isomers was between 15.6% and 19.9% (Supporting Information I, Table S3). In subjects where 2-OOH-3-(1,2-dioxane)-SQ was high, for example, subject 2 with 9.8 ng, we expected its precursor to be decreased in quantity among SQ-OOH isomers, however, 6-OOH-SQ in subject 2 represented 19.9% among SQ-OOH isomers, and was the highest percentage of 6-OOH-SQ among all subjects. On the other hand, SQ (chromatograms in Figure 8E,F) quantities were between 0.4 × 10^5^ and 2.7 × 10^5^ ng per sampled area (Figure 9B), and there was a clear correlation between the quantity of SQ and that of 2-OOH-3-(1,2-dioxane)-SQ per subject. Based on this observation and the relative stability of 2-OOH-3-(1,2-dioxane)-SQ, we concluded that 2-OOH-3-(1,2-dioxane)-SQ is formed on the skin from 6-OOH-SQ by photoinduced oxidation in the presence of ^3^O_2_, and can accumulate on the skin while new 6-OOH-SQ is being regenerated from SQ upon ^1^O_2_ oxidation. Here we confirmed that the newly identified compound, 2-OOH-3-(1,2-dioxane)-SQ is present in all analyzed SSLs from healthy individuals, making it the first evidence of the existence and quantification of this compound on the human skin. SQ-OOH, various lipid hydroperoxides, as well as certain lipid breakdown products such as MDA, have been often used as oxidative stress biomarkers [55], however, we demonstrated in this study the lability and instability of SQ-OOH isomers in conditions to which the skin is often exposed (i.e., photons and ^3^O_2_), hence, we suggest that 2-OOH-3-(1,2-dioxane)-SQ can be used as a more stable biomarker to better demonstrate the level of oxidative stress on the skin.

## 4. Conclusions

In this study, we identified a novel SQ secondary oxidation product as 2-OOH-3-(1,2-dioxane)-SQ, mainly in its cis configuration. We also confirmed the ^1^O_2_ induced generation of SQ-OOH on the skin from SQ and demonstrated that 2-OOH-3-(1,2-dioxane)-SQ is generated from 6-OOH-SQ upon its exposure to photons and in the presence of ^3^O_2_. The same oxidation pattern was observed in each SQ-OOH isomer to give similar (1,2-dioxane) species. We also developed an HPLC-MS/MS method for the analysis of 2-OOH-3-(1,2-dioxane)-SQ and were able to detect and quantify the compound in SSLs of six healthy volunteers. The obtained results indicate the possibility that the compound might accumulate on the skin. It is of great importance to further confirm and study in detail the oxidation mechanism proposed for the formation of the reported cyclic peroxides, as well as their effect on the skin and their abundance and role in subjects with altered skin conditions. Further oxidation of 2-OOH-3-(1,2-dioxane)-SQ (data not shown) showed more stability of the compound compared to when SQ-OOH isomers were further oxidized. This indicates a longer lifetime of 2-OOH-3-(1,2-dioxane)-SQ and longer periods of its contact with the skin, hence, we suggest that it can be used as a more reliable oxidative stress biomarker. Given its different nature that bears both a hydroperoxide and cyclic peroxide, 2-OOH-3-(1,2-dioxane)-SQ is expected to have a completely different effect on the skin. It is possible that other alkene bearing lipid species present on the skin can undergo the same mechanism reported in this study to give cyclic peroxide species, whose roles and nature are yet to be studied in depth.

## Figures and Tables

**Table 1:** Results of the ^13^C chemical shifts calculations carried out on Spartan 18 software.

	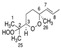 Cis 1A	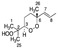 Trans 1A	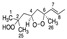 Cis 2A	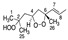 Trans 2A	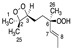 R 1B	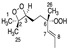 S 1B	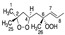 R 2B	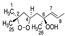 S 2B
RMS	0.96	1.85	9.55	10.05	4.23	4.31	10.72	10.35
Max absolute	1.80	3.40	21.00	22.00	7.20	5.90	31.60	30.20
Mean absolute	0.75	1.58	6.90	7.51	3.65	3.61	6.62	6.32

## Data Availability

Data is available in the article, supporting information I, and supporting information II.

## Supplementary Materials

The following are available online at https://www.mdpi.com/article/10.3390/antiox10111760/s1, Supporting Information I: Additional data (Figures S1–S4, Tables S1–S5), NMR assignments, and spectra (File type, PDF). Supporting Information II: Detailed Spartan’18 (Wavefunction Inc.) calculations (File type, Excel).
