# Supplementary material for: Novel Photoinduced Squalene Cyclic Peroxide Identified, Detected, and Quantified in Human Skin Surface Lipids
Source: Antioxidants (Basel). 2021 Nov 4;10(11):1760. doi: 10.3390/antiox10111760 (PMC8614752; doi:10.3390/antiox10111760)

Supporting information I

Novel photoinduced squalene cyclic peroxide identified, detected and quantified in human skin surface lipids

Saoussane Khalifa,[a] Masaru Enomoto,[b] Shunji Kato,[a] and Kiyotaka Nakagawa[a,*]

[a] Food and Biodynamic Chemistry Laboratory, Graduate School of Agricultural Science, Tohoku University, Sendai 980-8577, Japan

[b] Applied Bioorganic Chemistry Laboratory, Graduate School of Agricultural Science, Tohoku University, Sendai 980-8577, Japan

*Corresponding author:

Prof. K. Nakagawa

E-mail: kiyotaka.nakagawa.c1@tohoku.ac.jp

Content:

1. MRM conditions for the method optimized for 2-OOH-3-(1,2-dioxane)-SQ.
2. Q1 and PIS conditions for the analysis of individual SQ-OOH isomers' secondary oxidation products.
3. Percentage of individual SQ-OOH isomers among total SQ-OOH isomers in SSLs
4. Comparison of the PIS obtained form 6-OOH-SQ and 2-OOH-3-(1,2-dioxane)-SQ, Q1 scan of photooxidized 10-OOH-SQ.
5. Schemes and Q1 scan of the derivatization reactions carried out for the identification of 2-OOH-3-(1,2-dioxane)-SQ.
6. Q1 scan of the photooxidation of 10-OOH-SQ after 4 hours and 8 hours.
7. Q1 scan of the reaction between Mxp and the secondary oxidation product of each SQ-OOH isomer.
8. ^{1}H NMR and ^{13}C NMR spectral data, 1D and 2D spectra of the major and minor isomers of 2-OOH-3-(1,2-dioxane)-SQ.

1. MRM conditions for the method optimized for the analysis of 2-OOH-3-(1,2-dioxane)-SQ:

Table S1. MRM conditions for the method optimized for 2-OOH-3-(1,2-dioxane)-SQ.

	**Conditions**
	**MRM**
Source	ESI
Ion polarity	Positive
Declustering potential (V)	75
Entrance potential (V)	10
Temperature (°C)	600
Ion spray voltage (V)	5500
Curtain gas (psi)	20
Collision-activated dissociation gas (psi)	7
Collision energy (V)	25
Collision cell exit potential (V)	20
Ion source gas 1 (psi)	70
Ion source gas 2 (psi)	60

2. Q1 and PIS conditions for the analysis of individual SQ-OOH isomers' secondary oxidation products:

Table S2. Q1 and PIS conditions for the analysis of individual SQ-OOH isomers' secondary oxidation products.

	**Q1**	**MS/MS**
Source	ESI	ESI
Ion polarity	Positive	Positive
Mass range (***m/z***)	50-700	50-700
End plate offset (V)	-500	-500
Capillary (V)	4500	4500
Nebulizer (bar)	1.6	1.6
Dry gas (L/min)	6	6
Dry heater (°C)	180	180
Funnel 1RF (Vpp)	300	300
Funnel 2RF (Vpp)	400	400
Hexapole RF (Vpp)	400	400
isCID energy	0	0
Ion energy (eV)	3	3
Isolation mass (***m/z***)	300	300
Collision energy (eV)	14	30
Collision (RF) (Vpp)	400	400
Transfer time (μs)	70	70
Peak width	5	5

3. Percentage of individual SQ-OOH isomers among total SQ-OOH isomers in SSLs:

Table S3. Percentage of individual SQ-OOH isomers among total SQ-OOH isomers in SSLs.

Isomer	Subject1	Subject2	Subject3	Subject4	Subject5	Subject6
**11-OOH-SQ**	11.5%	10.7%	10.5%	11.6%	9.8%	11.9%
**7-OOH-SQ**	11.2%	11.9%	7.0%	10.2%	9.8%	12.1%
**10-OOH-SQ**	18.7%	21.2%	29.0%	24.5%	22.2%	23.1%
**6-OOH-SQ**	17.3%	19.9%	15.6%	18.0%	17.3%	18.1%
**3-OOH-SQ**	15.0%	14.9%	11.1%	14.2%	12.5%	15.2%
**2-OOH-SQ**	26.3%	21.4%	26.7%	21.5%	28.4%	19.6%

4. Comparison of the PIS obtained form 6-OOH-SQ and 2-OOH-3-(1,2-dioxane)-SQ, Q1 scan of photooxidized 10-OOH-SQ:

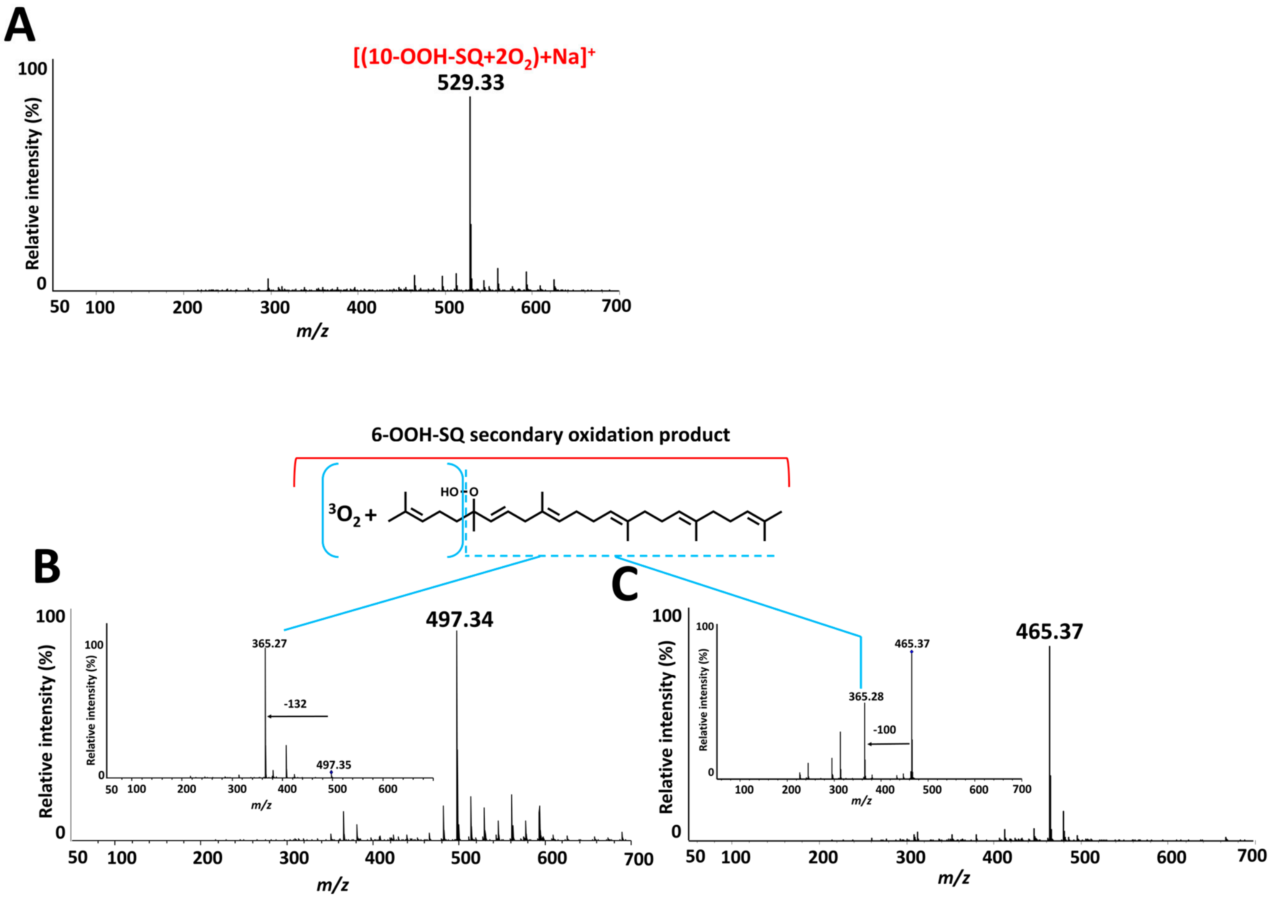

Figure S1. Q1 spectrum of photooxidized 10-OOH-SQ (4 hours) (A) and Q1 spectra and PIS of photooxidized 6-OOH-SQ (B) and unoxidized 6-OOH-SQ (C).

5. Schemes and Q1 scan of the derivatization reactions carried out for the identification of 2-OOH-3-(1,2-dioxane)-SQ:

- **Step1: reduction using Zinc dust**

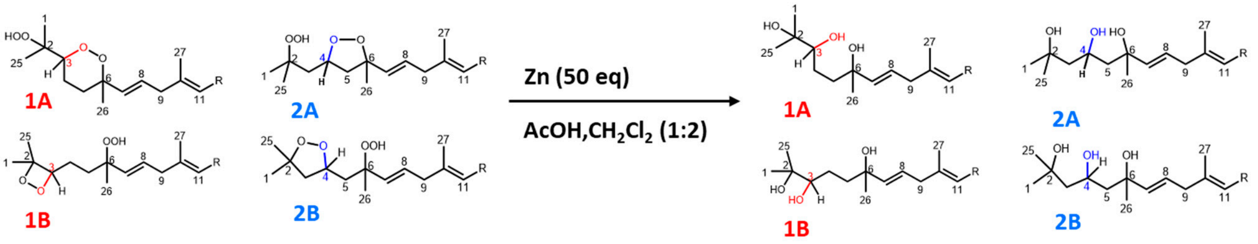

- **Step2: cleavage of vicinal alcohols using sodium periodate (NaIO$_4$)**

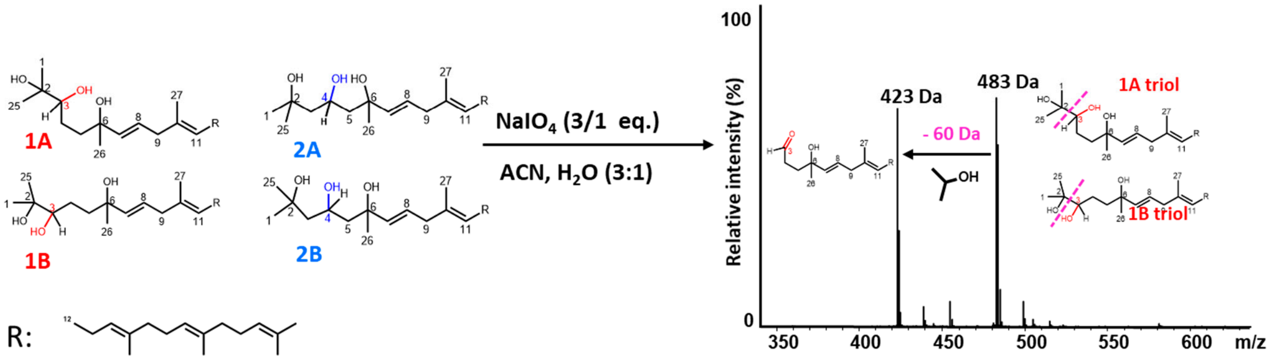

Figure S2. Derivatization reactions of SQ's main secondary oxidation product obtained from photooxidation of 6-OOH-SQ.

6. Q1 scan of the photooxidation of 10-OOH-SQ after 4 hours and 8 hours:

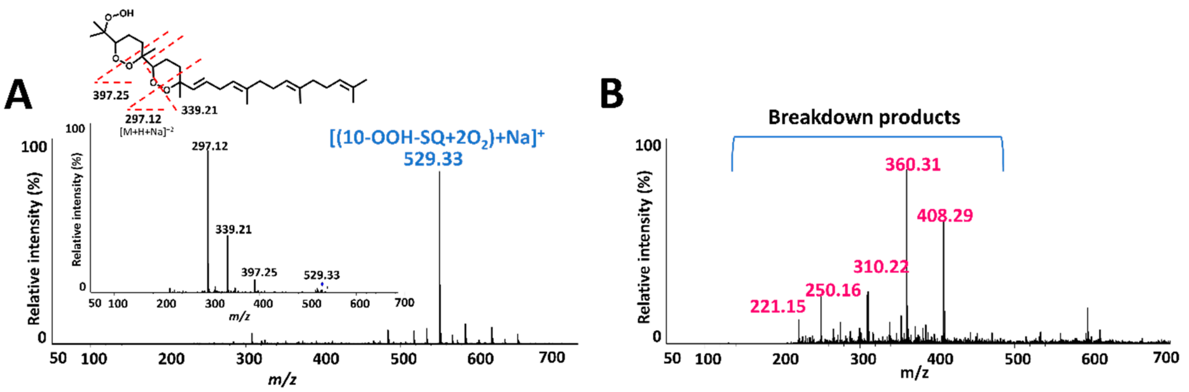

Figure S3. Formation of secondary oxidation product 2-OOH-3,7-di(1,2-dioxane)-SQ after 4 hours of oxidation of 10-OOH-SQ (Q1 + PIS) (A) and decomposition of the secondary oxidation product after 8 hours of oxidation (Q1) (B).

7. Q1 scan of the reaction between Mxp and the main secondary oxidation product of each SQ-OOH isomer.

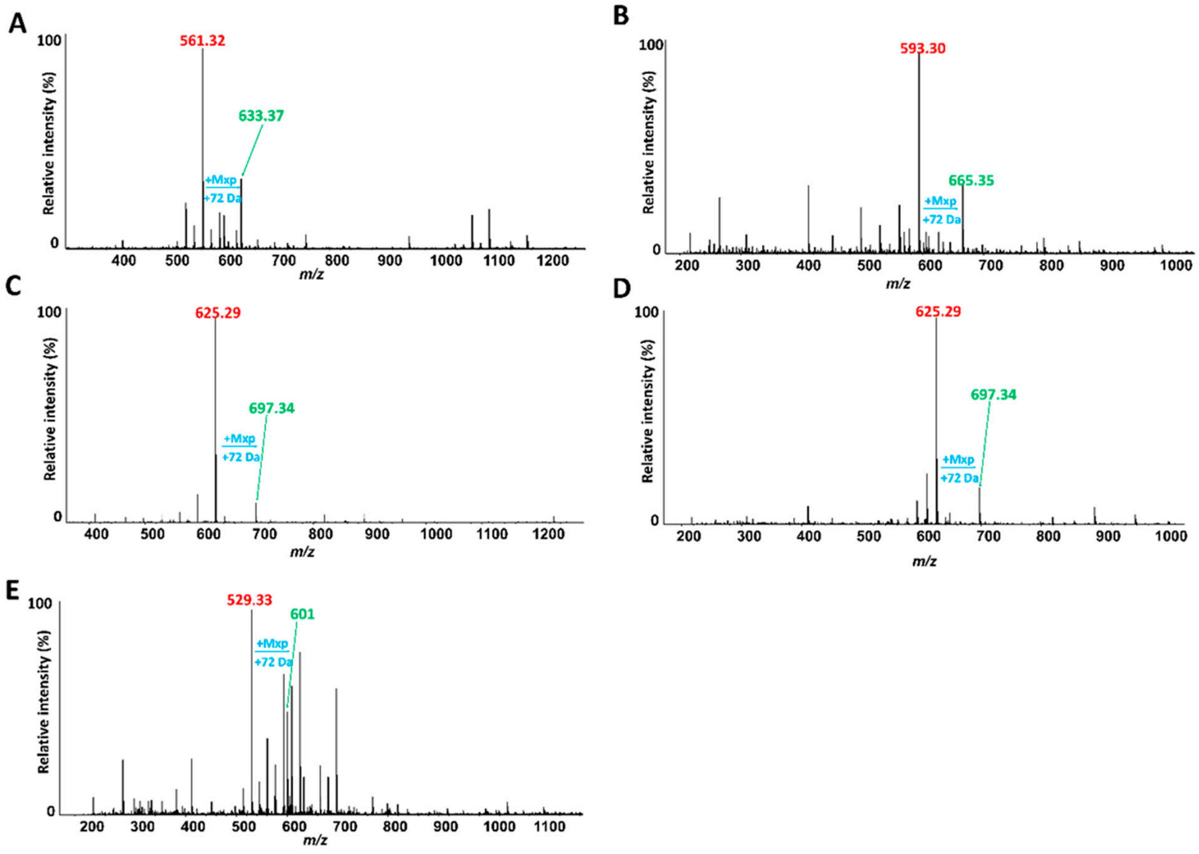

Figure S4. Mxp reaction with the crude sample of the secondary oxidation products obtained from the photooxidation of individual SQ-OOH isomers: (A) 11-OOH-SQ (containing mainly 11-OOH-SQ+3O_2), (B) 7-OOH-SQ (containing mainly 7-OOH-SQ+4O_2), (C) 3-OOH-SQ (containing mainly 3-OOH-SQ+5O_2), (D) 2-OOH-SQ (containing mainly 2-OOH-SQ+5O_2) and (E) 10-OOH-SQ (containing mainly 10-OOH-SQ+2O_2).

8. ^{1}H NMR and ^{13}C NMR spectral data of the major and minor isomers of 2-OOH-3-(1,2-dioxane)-SQ:

Table S4. ^{1}H NMR and ^{13}C NMR spectral data of the major isomer of 2-OOH-3-(1,2-dioxane)-SQ.

Position	1H NMR (CDCl3, 600 M Hz)	13C NMR (CDCl3, 150 M Hz)
**1**	1.21 (3H, s)	21.0
**2**	–	83.3
**3**	4.23 (1H, dd *J* = 10.5 3.3 Hz)	84.7
**4**	1.58-1.62 (1H, overlapping),<br>1.80-1.86 (1H, overlapping)	20.4
**5**	1.72-1.80 (1H, overlapping),<br>1.86-1.90 (1H, overlapping)	32.9
**6**	–	80.7
**7**	5.66 (1H, d, *J* = 15.6 Hz)	134.0
**8**	5.57 (1H, dt, *J* = 15.6, 7.2 Hz)	129.0
**9**	2.71 (2H, d, *J* = 6.6 Hz)	42.8
**10**	–	133.7
**11**	5.16-5.20 (1H, overlapping)	125.5
**12**	1.98-2.04 (2H, overlapping)	26.6, 26.7, 28.1, 28.2, 28.3, or 28.4
**13**	1.90-2.12 (2H, overlapping)	26.6, 26.7, 28.1, 28.2, 28.3, or 28.4
**14**	5.08-5.16 (1H, overlapping)	124.14, 124.22, or 124.37
**15**	–	135.2 or 134.9
**16**	1.90-2.12 (2H, overlapping)	26.6, 26.7, 28.1, 28.2, 28.3, or 28.4
**17**	1.90-2.12 (2H, overlapping)	26.6, 26.7, 28.1, 28.2, 28.3, or 28.4
**18**	5.08-5.16 (1H, overlapping)	124.14, 124.22, or 124.37
**19**	–	135.2 or 134.9
**20**	1.90-2.12 (2H, overlapping)	26.6, 26.7, 28.1, 28.2, 28.3, or 28.4
**21**	1.90-2.12 (2H, overlapping)	26.6, 26.7, 28.1, 28.2, 28.3, or 28.4
**22**	5.08-5.16 (1H, overlapping)	124.14, 124.22, or 124.37
**23**	–	131.3

**24**	1.68 (3H, s)	25.7
**25**	1.21 (3H, s)	21.0
**26**	1.20 (3H, s)	26.9
**27**	1.59 (3H, s)	16.1
**28**	1.60 (3H, s)	20.9 or 20.3
**29**	1.60 (3H, s)	20.9 or 20.3
**30**	1.60 (3H, s)	17.7

Table S5. ^{1}H NMR and ^{13}C NMR spectral data of the minor isomer of 2-OOH-3-(1,2-dioxane)-SQ.

**position**	**1H NMR (CDCl3, 600 M Hz)**	**13C NMR (CDCl3, 150 M Hz)**
**1**	1.24 (3H, s)	20.9
**2**	—	83.2
**3**	4.22 (1H, dd, *J* = 10.5, 3.3 Hz)	84.7
**4**	1.66-1.72 (1H, overlapping), 1.84-1.90 (1H, overlapping)	20.4
**5**	1.72-1.80 (1H, overlapping) 2.02-2.06 (1H, overlapping)	32.7
**6**	—	79.9
**7**	5.44 (1H, d, *J* = 16.2 Hz)	134.1
**8**	5.68 (1H, dt, J = 15.6, 7.2 Hz)	130.4
**9**	2.69 (2H, d, *J* = 6.6 Hz)	42.8
**10**	—	133.2
**11**	5.16-5.20 (1H, overlapping)	125.7
**12**	1.98-2.04 (2H, overlapping)	26.6, 26.7, 28.1, 28.2, 28.3, or 28.4
**13**	1.90-2.12 (2H, overlapping)	26.6, 26.7, 28.1, 28.2, 28.3, or 28.4
**14**	5.08-5.16 (1H, overlapping)	124.10, 124.22, or 124.37
**15**	–	135.2 or 134.9
**16**	1.90-2.12 (2H, overlapping)	26.6, 26.7, 28.1, 28.2, 28.3, or 28.4

**17**	1.90-2.12 (2H, overlapping)	26.6, 26.7, 28.1, 28.2, 28.3, or 28.4
**18**	5.08-5.16 (1H, overlapping)	124.10, 124.22, or 124.37
**19**	–	135.2 or 134.9
**20**	1.90-2.12 (2H, overlapping)	26.6, 26.7, 28.1, 28.2, 28.3, or 28.4
**21**	1.90-2.12 (2H, overlapping)	26.6, 26.7, 28.1, 28.2, 28.3, or 28.4
**22**	5.08-5.16 (1H, overlapping)	124.10, 124.22, or 124.37
**23**	–	131.3
**24**	1.68 (3H, s)	25.7
**25**	1.28 (3H, s)	21.2
**26**	1.48 (3H, s)	20.9
**27**	1.59 (3H, s)	16.1
**28**	1.60 (3H, s)	20.9 or 20.3
**29**	1.60 (3H, s)	20.9 or 20.3
**30**	1.60 (3H, s)	17.7

^{1}H NMR

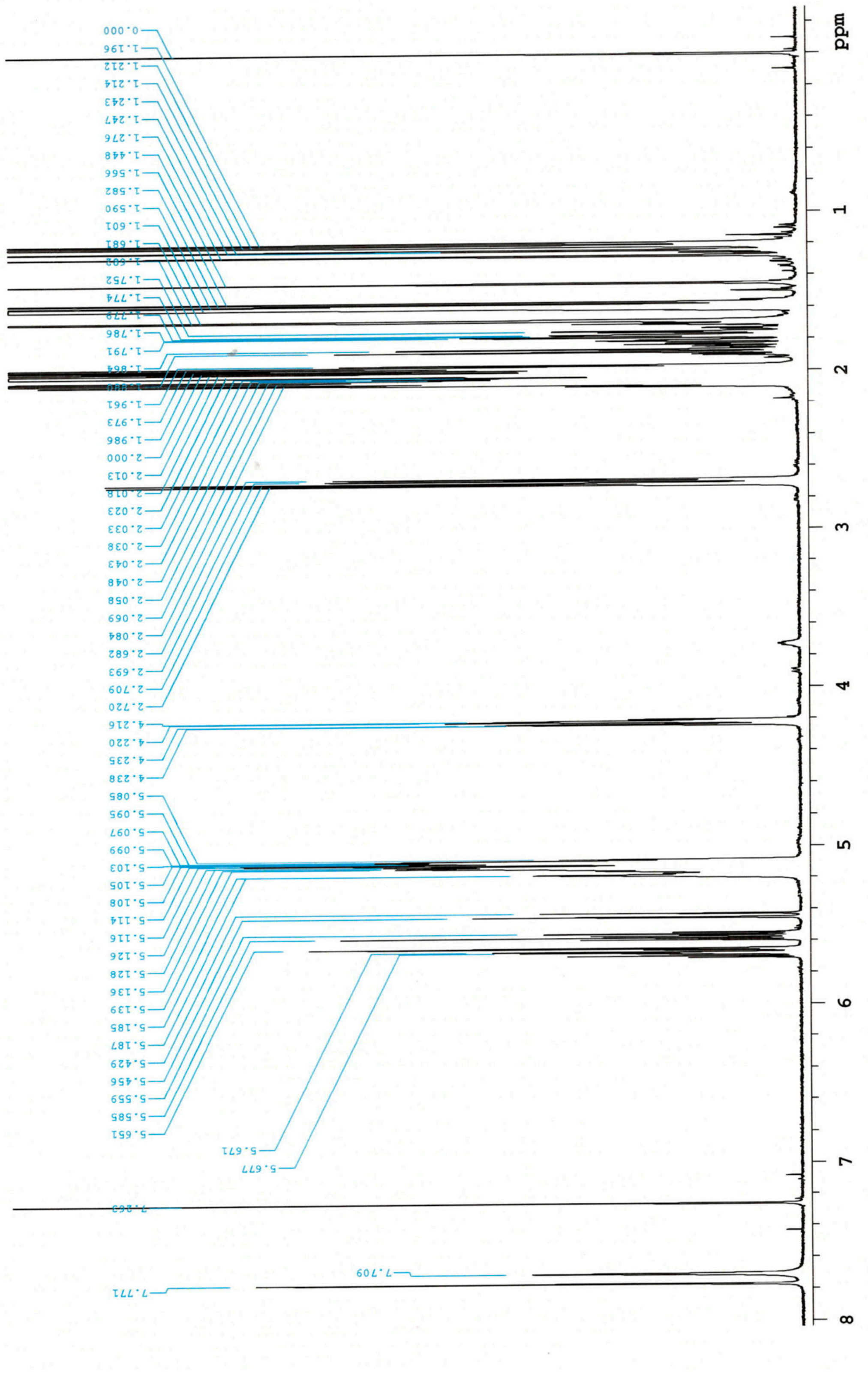

0.000
1.196
1.212
1.214
1.243
1.247
1.276
1.448
1.566
1.582
1.590
1.601
1.681
1.752
1.774
1.778
1.786
1.791
1.961
1.973
1.986
2.000
2.013
2.018
2.023
2.033
2.038
2.043
2.048
2.058
2.069
2.084
2.682
2.693
2.709
2.720
4.216
4.222
4.235
4.238
5.085
5.095
5.097
5.099
5.103
5.105
5.108
5.114
5.116
5.126
5.128
5.136
5.139
5.185
5.187
5.429
5.456
5.559
5.585
5.651
5.671
5.677
7.709
7.771
ppm
1
2
3
4
5
6
7
8

^{13}C NMR

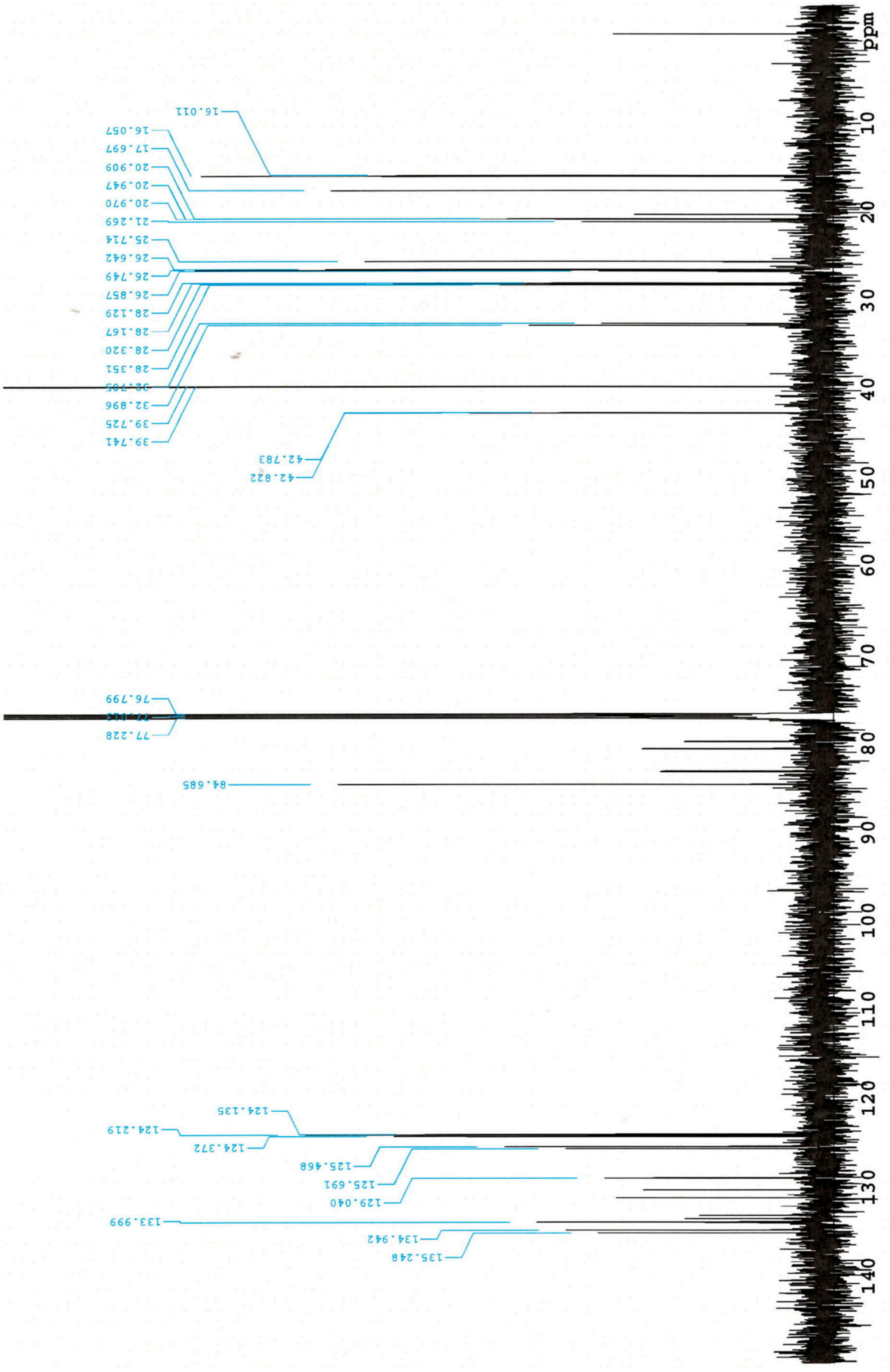
ppm
10
20
30
40
50
60
70
80
90
100
110
120
130
140
16.011
16.057
17.697
20.909
20.947
20.970
21.269
25.714
26.642
26.749
26.857
28.129
28.167
28.320
28.351
32.896
39.725
39.741
42.783
42.822
76.799
77.228
84.685
124.135
124.219
124.372
125.468
125.691
129.040
133.999
134.942
135.248

DEPT

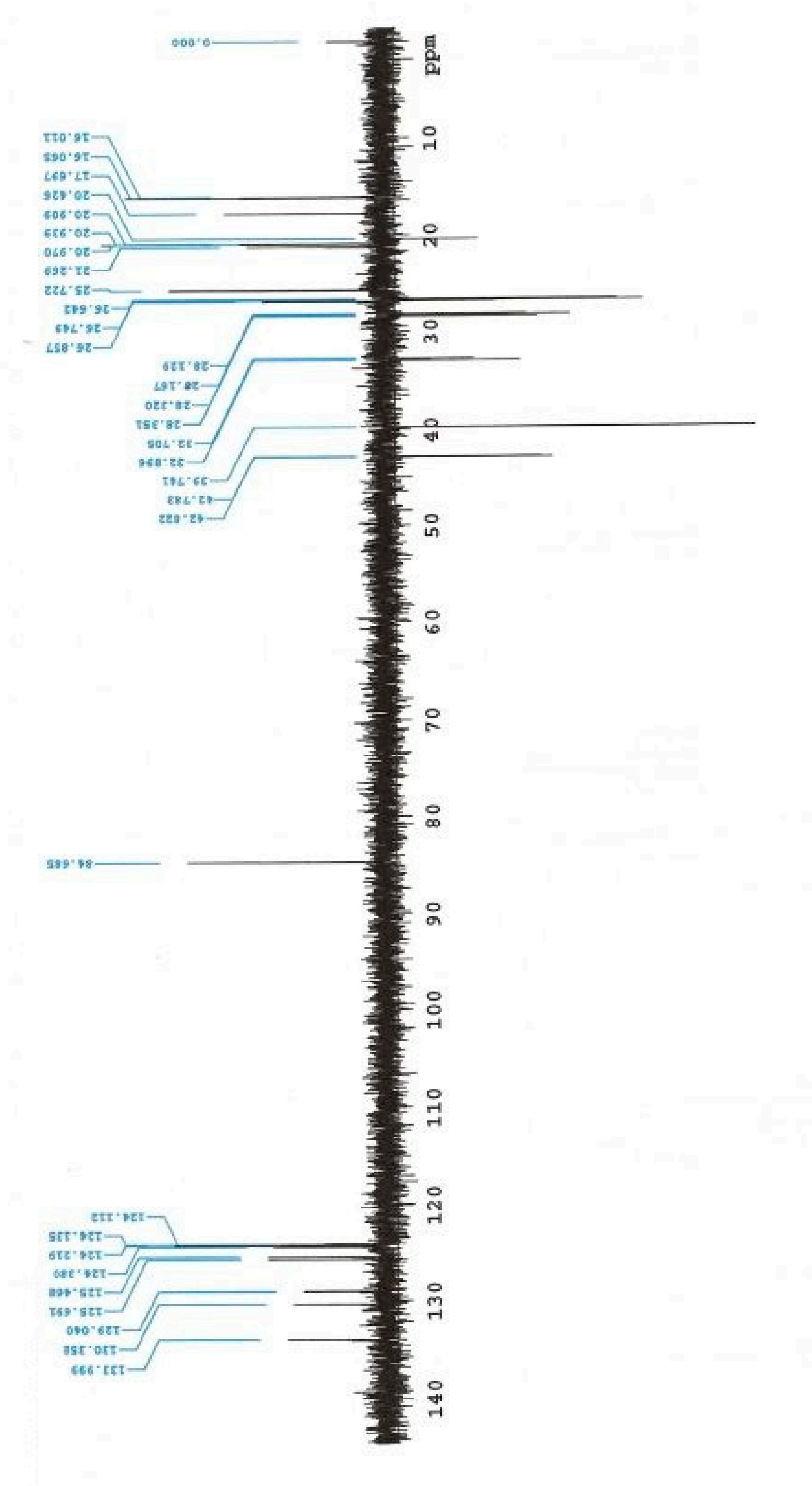
ppm
0.000
16.011
16.065
17.697
20.626
20.909
20.939
20.970
21.269
25.722
26.643
26.749
26.857
28.139
28.167
28.320
28.351
32.705
32.896
39.741
42.783
42.832
84.665
124.112
124.135
124.319
124.380
125.468
125.691
129.060
130.358
133.999
10
20
30
40
50
60
70
80
90
100
110
120
130
140

HMBC

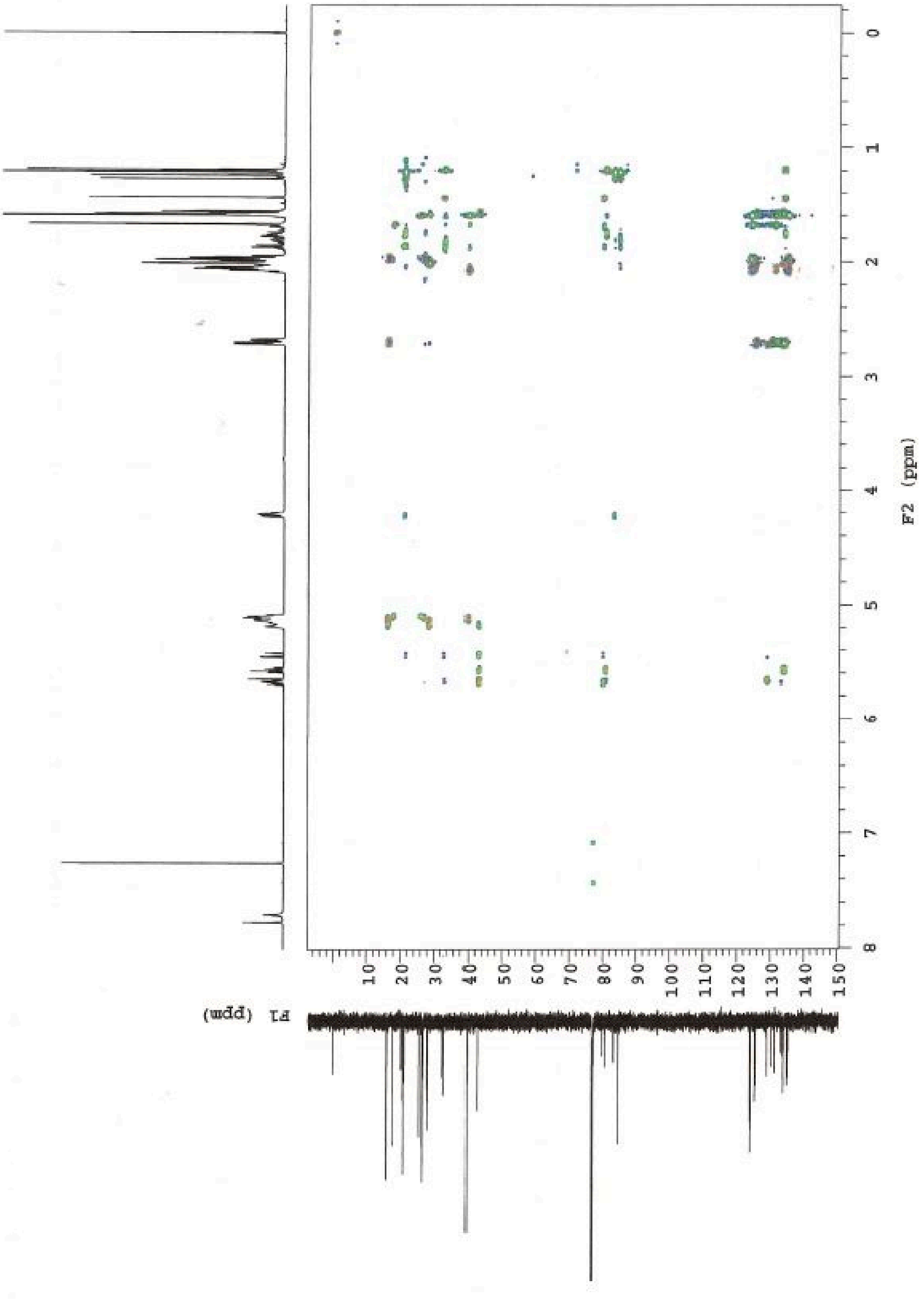

HSQC

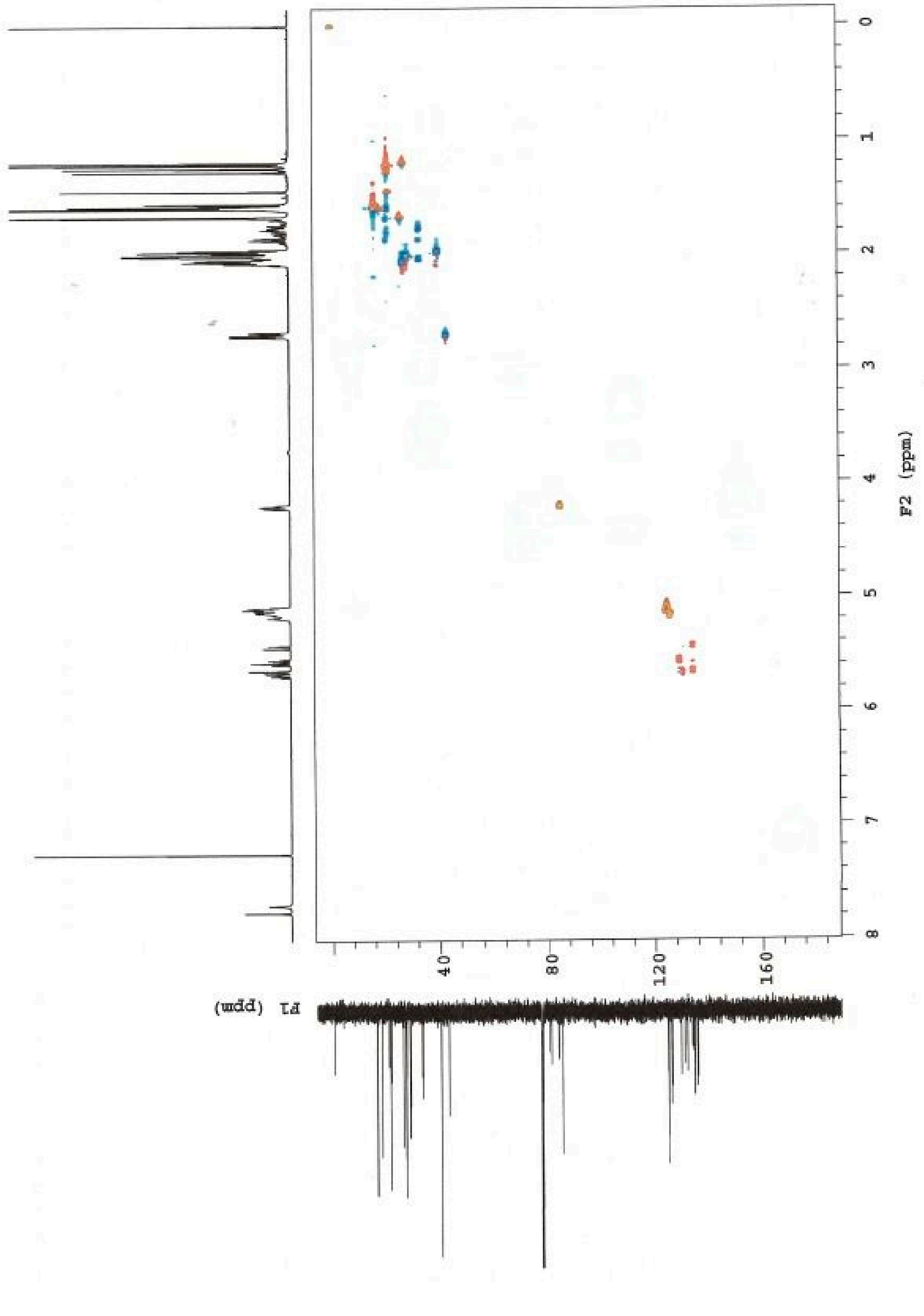

NOESY

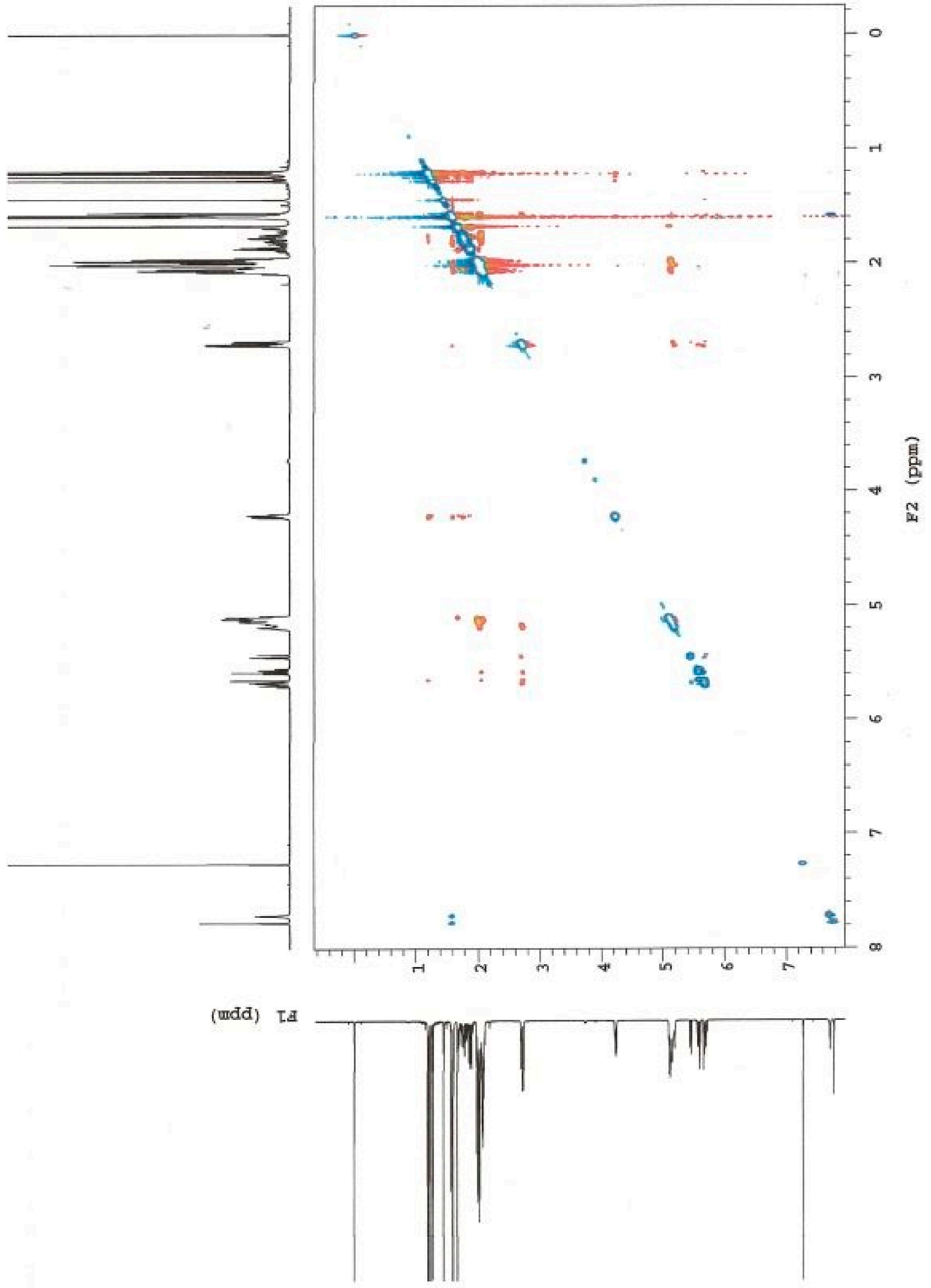

COSY

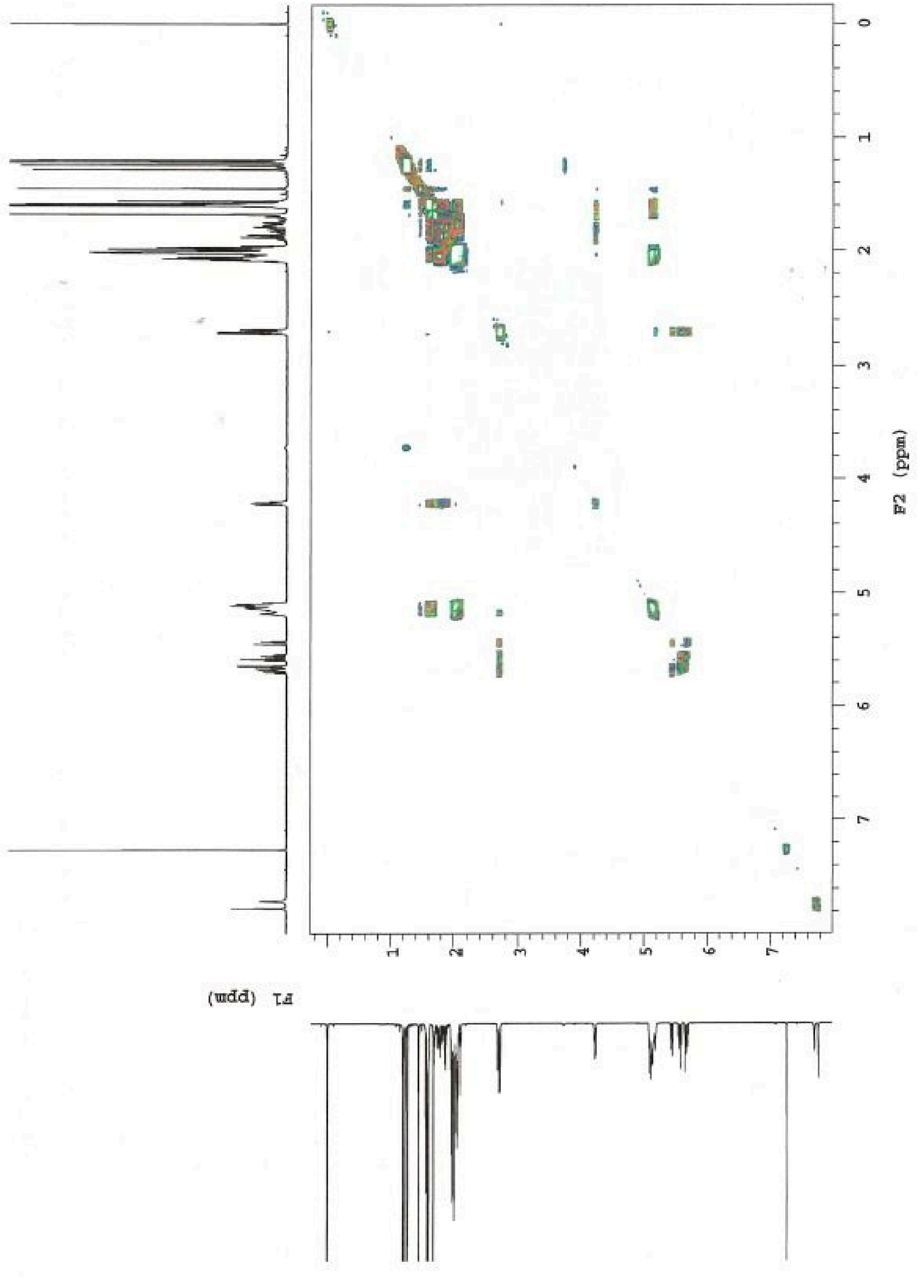

Supplement: Supplementary file 1 [file antioxidants-10-01760-s001.zip › Supporting information 1.pdf]
